# Breaking the Limitations of Sulfur Redox Kinetics by Accelerated Li^+^-Desolvation in Lithium–Sulfur Batteries

**DOI:** 10.1007/s40820-026-02232-6

**Published:** 2026-05-27

**Authors:** Tan Wang, Zhenhua Wang, Xiaotian Gao, Zhe Bai, Wanning Liu, Yu Bai, David Rooney, Kening Sun

**Affiliations:** 1 Beijing Key Laboratory of Green Hydrogen and Fuel Cells, School of Chemistry and Chemical Engineering, Beijing Institute of Technology, Beijing, 100081 People’s Republic of China; 2 School of Chemistry and Chemical Engineering, Queen’s University Belfast, Belfast, Northern Ireland BT9 5AG UK

**Keywords:** Desolvation energy barrier, Single-atom catalysts, Catalyst desolvation, Sulfur redox kinetics, Lithium−sulfur batteries

## Abstract

The practical deployment of lithium–sulfur batteries (LSBs) is fundamentally limited by the sluggish stepwise sulfur redox kinetics. However, current design philosophies remain heavily constrained by the conventional “adsorption-catalysis” strategy, often overlooking the crucial rate-limiting kinetic obstacle of the high Li^+^ desolvation energy barrier. This sluggish Li^+^ desolvation process imposes a severe kinetic penalty on polysulfide conversion, thereby depressing electrochemical stability. Herein, we propose a catalyst desolvation strategy utilizing a Ce single-atom catalyst to promote the Li^+^ desolvation process, thereby enhancing the redox conversion of polysulfides. Results indicate that the catalyst desolvation strategy increases the proportion of contact ion pairs and aggregates, reduces the Li^+^ desolvation energy barrier, and stabilizes the lithium anode/electrolyte interface. Consequently, the accelerated Li^+^ desolvation facilitates rapid sulfur redox kinetics, thereby realizing stable cycling in LSBs with a low decay rate of 0.036% per cycle over 1700 cycles at 1 C. This work confirms the significant impact of Li^+^ desolvation and provides a new solution for achieving efficient conversion of polysulfides in LSBs.

**Supplementary Information:**

The online version contains supplementary material available at 10.1007/s40820-026-02232-6.

## Highlights

- A catalyst desolvation strategy via phosphorus-modulated Ce single-atom catalysts is proposed to reduce the Li^+^ desolvation barrier and accelerate reaction kinetics.
- The Ce-*f* orbital achieves a maximized overlap with the S-*p* orbital, and this strengthened* f*-*d*-*p* orbital hybridization effectively inhibits the diffusion of polysulfide anions through the interlayer.
- Synergizing accelerated Li^+^ desolvation with suppressed polysulfide shuttling, the batteries demonstrate ultrastable cycling with an ultralow decay rate of 0.036% per cycle over 1700 cycles.

**Supplementary Information:**

The online version contains supplementary material available at 10.1007/s40820-026-02232-6.

## Graphical abstract

**Figure:**
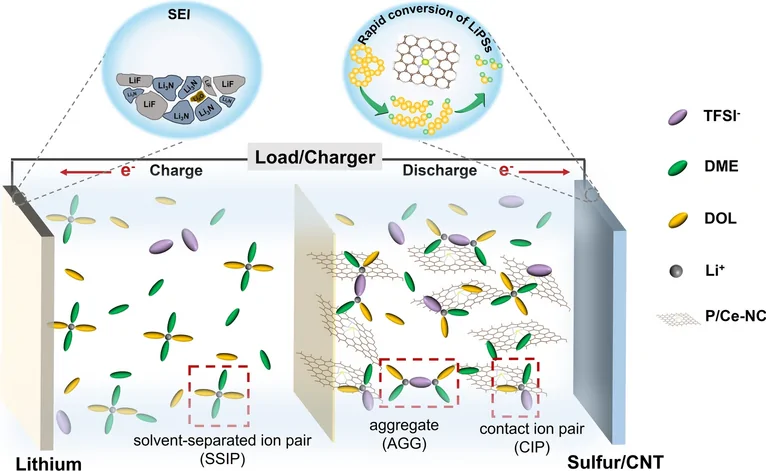

## Introduction

Lithium–sulfur batteries (LSBs) with high theoretical specific capacities for sulfur cathode (1675 mAh g^−1^) and lithium anode (3860 mAh g^−1^) are regarded as promising next-generation batteries [1–3]. However, their practical implementation faces major challenges. Key obstacles include sluggish stepwise sulfur conversion kinetics, the notorious shuttling effect of lithium polysulfides (LiPSs), and uncontrollable dendrite growth on the Li anode, all of which result in unsatisfactory electrochemical stability [4–6]. Prevailing strategies to address such issues focus on chemically suppressing LiPSs shuttling and enhancing their conversion kinetics [7–10]. Various non-metallic/metal-based catalysts have been developed for this purpose, including metal oxides [11, 12], sulfides [13, 14], and nitrides [15]. However, these conventional “adsorption-catalysis” strategies often overlook the crucial rate-limiting step of the Li^+^-solvents desolvation.

In most liquid electrolytes, the Li^+^ desolvation process is inherently sluggish, typically attributed to a solvation structure dominated by solvent-separated ion pair (SSIP) [16]. To overcome this challenge, many strategies focus on modulating the Li^+^ solvation structure by selecting solvents with low desolvation energy, adding desolvation promoters, and elevating salt concentration [17–20]. These approaches can modulate the Li^+^ solvation structure from a SSIP-dominated state to a state dominated by contact ion pairs (CIP) and aggregation ion pairs (AGG) [21–23]. However, excessive additives or high salt concentrations can severely compromise the ionic conductivity due to increased viscosity, consequently impeding the overall conversion efficiency of LiPSs.

Fundamentally, the Li^+^-solvents desolvation can be regarded as a decomposition reaction with a high energy barrier, and thus the sluggish Li^+^ desolvation kinetics at the interface significantly impede the redox conversion of polysulfides [24]. The desolvation process involves overcoming a substantial energy barrier to break the strong Li^+^-solvent coordination bonds [25–27]. Conceptually, the bond-breaking process is fundamentally analogous to the intermediate adsorption process in a heterogeneous catalytic reaction, implying that the desolvation barrier can be effectively lowered by a catalyst. The specific active sites on the catalyst surface could adsorb solvent molecules to weaken the Li^+^-solvent interaction, thereby effectively accelerating the Li^+^ desolvation process. Therefore, employing catalysts with a specific electronic structure offers a promising strategy to catalyze this desolvation step [28].

Single-atom catalysts (SACs) achieve ~100% atomic utilization and exceptional catalytic activity, functioning as selective kinetic promoters to drive conversion reactions [29, 30]. Isolated active center atoms in SACs could effectively weaken the Li^+^-solvent attraction within the solvation sheath, thereby lowering the desolvation energy barrier and accelerating reaction kinetics [31]. Unlike the broadband characteristics of *d* or *p* orbitals, the *f* orbitals of rare earth elements feature unique narrow-band properties [32, 33]. Furthermore, their tunable 4*f* energy levels and strong spin-orbit coupling offer unique potential for modulating the electron structure of the active center atom [34–36]. Among various rare earth elements, cerium (Ce) serves as an abundant and versatile electronic orbital carrier. It possesses unique, highly localized, and partially occupied 4*f* orbitals. Additionally, variable oxidation states and flexible coordination numbers are recognized as the distinctive features of Ce [37, 38]. Regulating the local microenvironment of the central atom via heteroatoms holds the promise of a significant performance leap for Ce-SACs. Therefore, we expect to utilize heteroatom-regulated Ce-SACs to fundamentally understand the driving forces of the desolvation process and the interfacial solvation structure changes induced by the active sites.

**Fig. 1:**
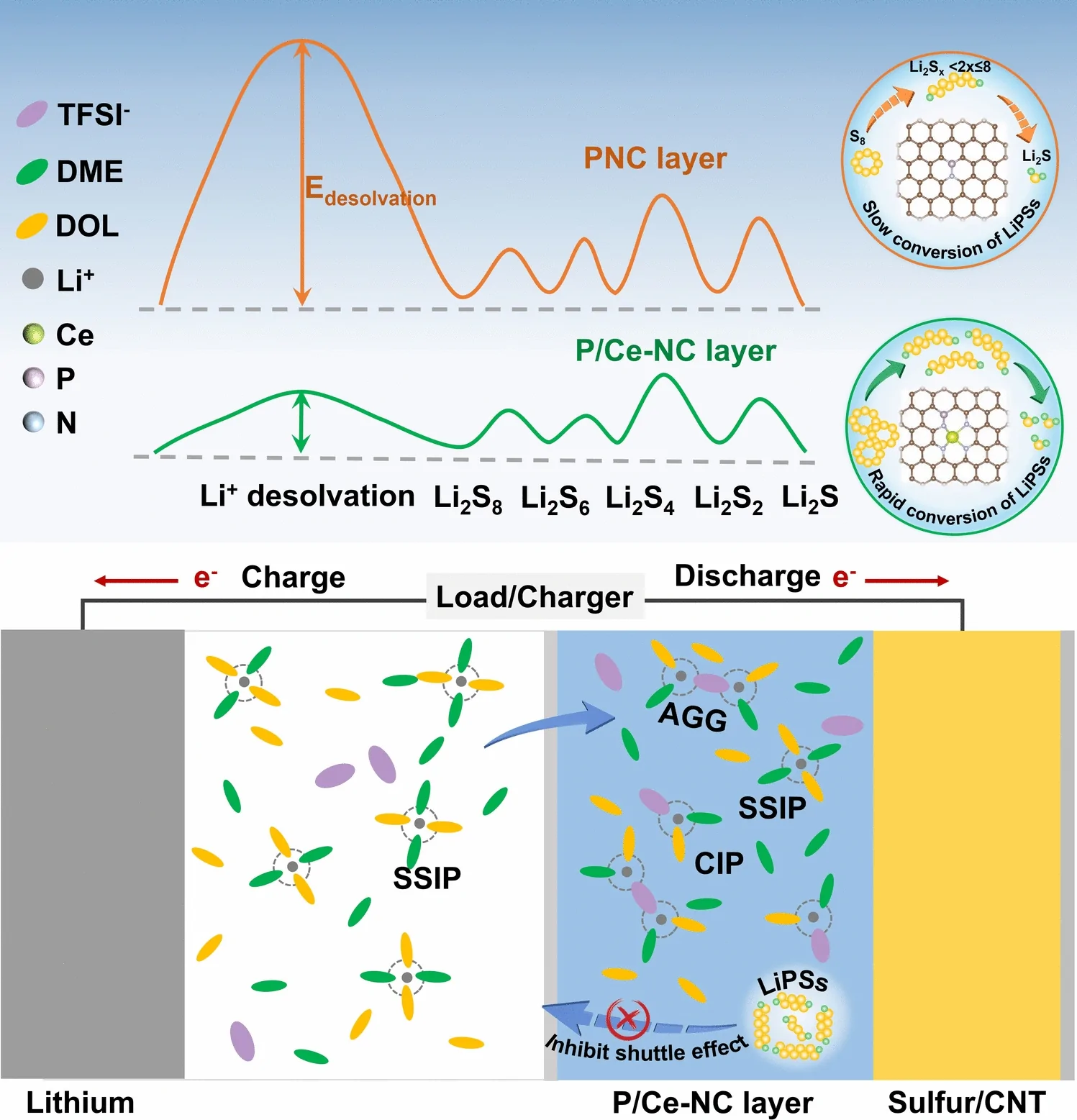
Schematic of catalyst desolvation by P/Ce-NC catalyst layer

As shown in Fig. 1, the Li^+^ desolvation represents a decomposition reaction characterized by a high energy barrier, rendering it the rate-limiting step for polysulfide conversion. Benefiting from the catalyst desolvation strategy, the Li^+^ desolvation process is boosted by employing a catalyst layer composed of phosphorus-modulated Ce SACs. Specifically, phosphorus incorporated into the second coordination sphere of the Ce−N_4_ site precisely modulates the electron occupancy of the* f*_*y3x*_^*2*^ and* f*_*z*_^*3*^ orbitals of Ce, thereby weakening the interaction between Li^+^ and solvent molecules. Consequently, the P/Ce-NC promotes the reorganization of the Li^+^ solvation structure and formation of more CIP and AGG, thereby lowering the Li^+^-solvent desolvation energy barrier. Moreover, the Ce-*f* orbital achieves a maximized overlap with the S-*p* orbital, and this strengthened *f-d-p* orbital hybridization further effectively inhibits the diffusion of polysulfide anions through the interlayer. The accelerated Li^+^-solvent desolvation enhances the redox conversion of polysulfides. This catalyst desolvation strategy opens a new avenue toward enhanced sulfur redox kinetics in LSBs.

## Experimental Section

In a typical synthesis, 64.5 mg of Ce(NO_3_)_3_∙6H_2_O was dissolved in 200 mL of methanol and heated to 60 °C. Then, 84 μL phytic acid (PA, 50% (w/w) H_2_O solution) was added, and the mixture was stirred for 2 h to form stable PA-Ce complexes through the strong chelating capability of phosphate groups. Subsequently, a methanol solution containing 2.5 g of Zn(NO_3_)_2_·6H_2_O (dissolved in 200 mL of methanol) and 3.0 g of 2-methylimidazole (dissolved in 200 mL of methanol) was sequentially added after being stirred for 2 h. Stirred the mixed solution at 60 °C for 24 h. Then, centrifuged, washed, and dried, and named P/Ce-ZIF8. Subsequently, the sample was pyrolyzed at 1000 °C for 1 h under a continuous Ar flow. Finally, the single-atom catalyst P/Ce-NC was obtained. It is crucial that the strong coordination provided by PA effectively prevents the migration, aggregation, or loss of Ce atoms during harsh heat treatment processes. For comparison, the same procedure was performed without the addition of PA. Notably, no Ce species could be successfully incorporated or detected in the final catalyst in the absence of the PA anchoring effect (Table S1). The PNC was synthesized using the same method but omitting Ce (NO_3_)_3_∙6H_2_O. Similarly, the P/Ce@NC was prepared with the addition of 129 mg of Ce (NO_3_)_3_∙6H_2_O, keeping all other steps identical.

## Results and Discussion

### Synthesis and Structural Characterization of P/Ce-NC Catalyst

**Fig. 2:**
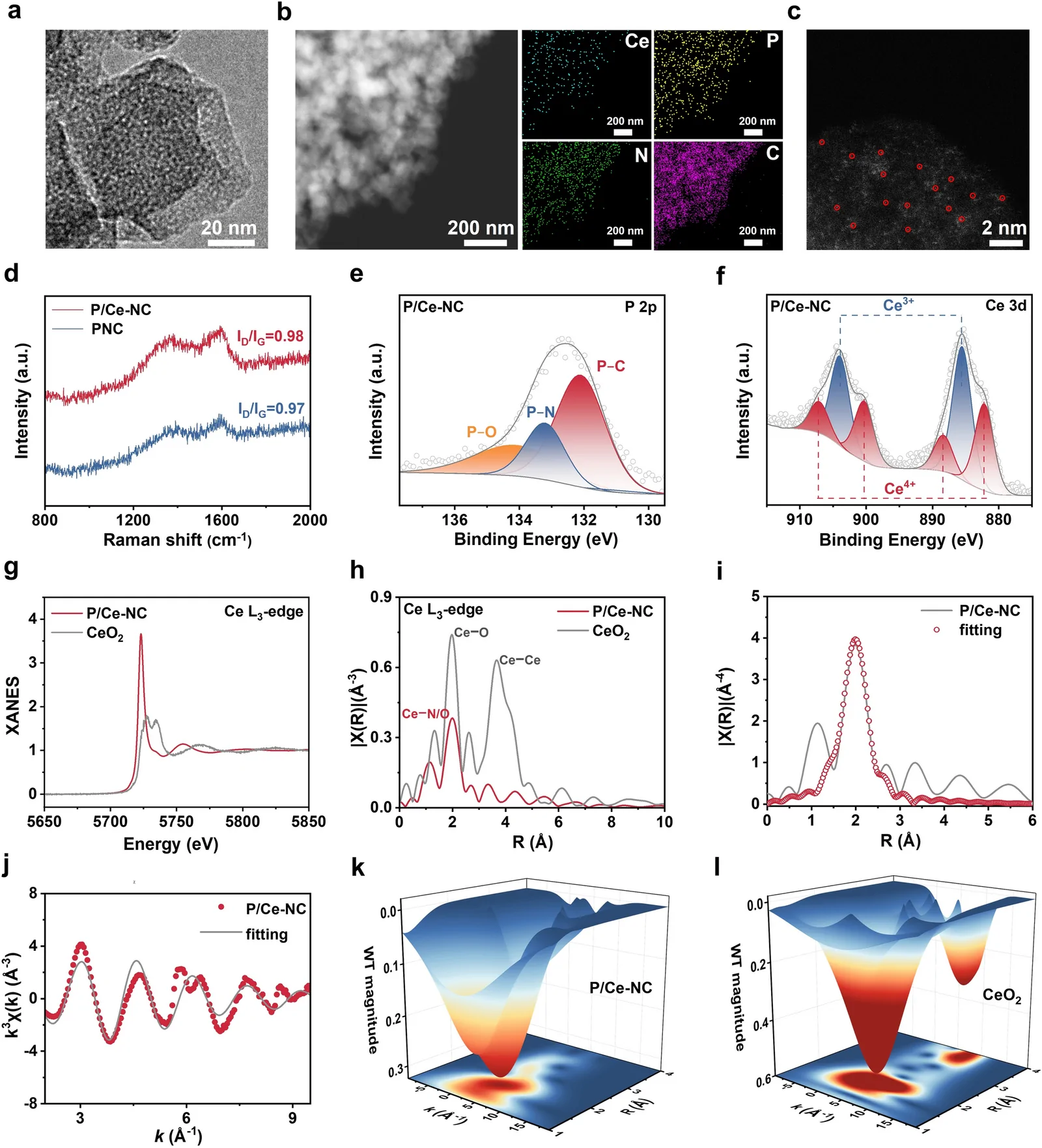
**a** TEM image, **b** EDS elemental mapping images, and **c** HAADF image of P/Ce-NC. **d** XRD patterns. XPS spectra of **e** p 2*p* and **f** Ce 3*d* in the P/Ce-NC. **g** Ce L_3_-edge XANES, **h** FT-EXAFS spectra, **i** EXAFS *R*-space fitting curves, **j** EXAFS *k*-space fitting curves of P/Ce-NC. Wavelet transform of **k** P/Ce-NC, **l** CeO_2_

Ce(NO_3_)_3_·6H_2_O and phytic acid solution were added to methanol, followed by the sequential addition of Zn(NO_3_)_2_·6H_2_O and dimethylimidazole. The product was named P/Ce-ZIF8, and the X-ray diffraction (XRD) analysis is shown in Fig. S1. Subsequently, the isolated Ce single atoms were anchored onto the ZIF8-derived N-doped carbon after pyrolysis at 1000 °C. Phytic acid acted as a phosphorus source to construct a second coordination sphere around the Ce active center. The Ce content of the produced P/Ce-NC sample measured by ICP-OES was 5.30 wt% (Table S2). The powder X-ray diffraction (XRD) patterns of the P/Ce-NC and PNC show two peaks at approximately 25° and 43.5°, referring to the (002) and (101) facets of graphite, respectively (Fig. S2). Scanning electron microscopy (SEM) was employed to characterize the morphologies of P/Ce-NC and PNC, with the corresponding results presented in Fig. S3. No Ce-containing nanoparticles (such as Ce and/or CeP) were detected in P/Ce-NC and PNC samples via transmission electron microscopy (TEM) and high-resolution transmission electron microscopy (HRTEM) in Fig. S4. As depicted in Fig. 2a, P/Ce-NC depicts a rhombic dodecahedron shape with a diameter of approximately 60 nm. As shown in Fig. 2b, the energy-dispersive X-ray spectroscopy (EDS) mapping confirms the homogeneous distribution of Ce, P, N, and C throughout the P/Ce-NC material. At the same time, the high-angle annular dark-field scanning TEM (HAADF-STEM) test can also directly observe the homogeneous dispersion of Ce single atoms (highlighted with red circles) in Figs. 2c and S5. The surface area (879.95 m^2^ g^−1^) and the total pore radius (2.66 nm) are tested by the Brunauer−Emmett−Teller (BET) test in Fig. S6. The I_D_/I_G_ ratio in Raman spectroscopy of P/Ce-NC (0.98) and PNC (0.97) indicates a similar degree of graphitization (Fig. 2d).

The elemental chemical environment of P/Ce-NC was analyzed by X-ray photoelectron spectroscopy (XPS). Curve fitting of the N 1*s* XPS spectrum reveals the presence of four distinct nitrogen species in Fig. S7, assigned as pyridinic N (398.6 eV), pyrrolic N (400.2 eV), graphitic N (401.5 eV), and oxidized N (403.8 eV) [39]. The P 2*p* XPS spectrum of P/Ce-NC can be fitted with three peaks located at 132.2, 133.3, and 134.2 eV, attributable to P−C, P−N, and P−O groups, respectively (Fig. 2e). This further confirms the successful incorporation of P. The Ce 3*d* XPS spectrum can be deconvoluted into six peaks. Among these, the peaks at 903.96 and 885.53 eV correspond to Ce^3+^, while the other peaks represent Ce^4+^ [40] (Fig. 2f).

X-ray absorption fine structure (XAFS) measurements were conducted to reveal the local atomic structure of the Ce center in the P/Ce-NC. As illustrated in Fig. 2g, unlike the characteristic double peak of CeO_2_ at 5727.22 and 5734.19 eV, the Ce L_3_-edge of P/Ce-NC displays a distinct single peak at 5723.00 eV. This reveals the valence state of the Ce species in P/Ce-NC is lower than +4. Subsequently, the X-ray absorption near-edge structure (XANES) spectra at the Ce L_3_-edge of P/Ce-NC exhibit a single peak corresponding to Ce−N/O bonds at 1.99 Å (Fig. 2h). The bond length of this peak is slightly longer than that of the Ce−O bond (1.96 Å), indicating the presence of Ce−N bonds. Notably, no peak associated with the Ce−Ce bond is detected at 3.65 Å, which is consistent with the aforementioned HAADF-STEM results. Furthermore, EXAFS fitting reveals that each isolated Ce is coordinated by four N and four O (Fig. 2i, j, Table S3). A possible mechanism is that Ce has a high coordination ability and strong affinity for oxygen, which makes it easy to absorb O_2_ from the atmosphere. The wavelet transform (WT) EXAFS is shown in Fig. 2k, l where the K-value corresponding to Ce−N/O scattering is approximately 3.2 Å^−1^. It is worth noting that no other obvious absorption peaks are observed in the spectra.

### Mechanism of Accelerated Li^+^-Solvents Desolvation Process by P/Ce-NC Catalyst Layer

**Fig. 3:**
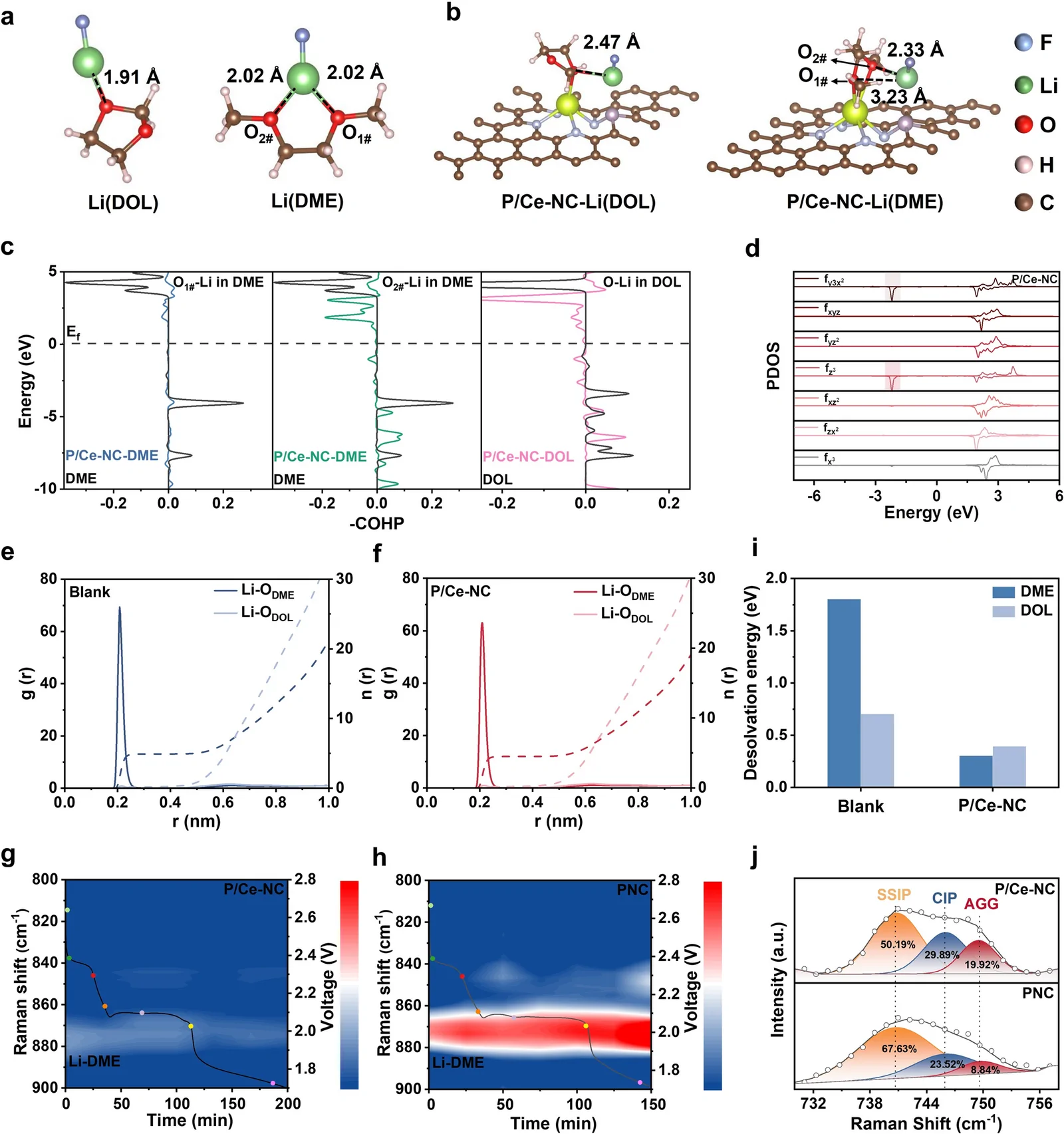
Geometric configurations of Li^+^ solvation structure **a** in DOL/DME, and **b** on P/Ce-NC. **c** Bonding state change of Li−O in DME/DOL. **d** PDOS of the Ce-*f* orbitals in P/Ce-NC. The coordination number of Li^+^−O in the blank electrolyte **e** without and **f** with P/Ce-NC. *In situ* Raman spectra with **g** P/Ce-NC and **h** PNC catalyst layer. **i** Li^+^ desolvation energy on different substrates. **j** Raman spectra of the various catalyst layers after one cycle

Due to the four O atoms on the surface of P/Ce-NC derived from atmospheric adsorption, these O atoms undergo desorption and Li_2_S_6_ adsorption during the electrochemical process. Therefore, the Ce−N_4_ model was used for calculations [38, 41]. Density functional theory (DFT) calculations reveal the mechanism by which P/Ce-NC regulates Li⁺-solvent interactions. Figure 3a, b shows the bond-length changes upon introducing the P/Ce-NC catalyst. In the P/Ce-NC-Li(DOL) system, the Li−O bond length increases from 1.91 to 2.47 Å, and in the P/Ce-NC-Li(DME) system, the Li−O_1#_/Li−O_2#_ bond lengths increase from 2.02 to 3.23/2.33 Å, respectively. This indicates the P/Ce-NC can weaken the Li⁺-solvent interactions, thus promoting the alteration of the solvation structure.

Crystal orbital Hamilton population (COHP) analysis quantifies this effect. The positive signals correspond to bonding states, and negative signals correspond to antibonding states (Fig. 3c). The integrated pCOHP (IpCOHP) value for P/Ce-NC of Li−O_1#_ in DME, Li−O_2#_ in DME, and Li−O in DOL are 0.02, 0.12, and 0.06, respectively, confirming reduced binding strength (Table S4). These values are significantly lower than the pure solvent system, indicating that the P/Ce-NC substantially weakens the interaction between Li^+^ and solvent molecules. Density of states (DOS) calculations further elucidate the electronic origin (Figs. 3d and S8). Phosphorus incorporation into the second coordination sphere of Ce–N_4_ increases the electron occupancy of the Ce *f*_*y3x*_^*2*^ and* f*_*z*_^*3*^ orbitals. This electronic reconfiguration enhances the interaction between the Ce and O atoms of the solvent, consequently weakening the Li^+^-solvent interaction and accelerating the desolvation process.

Molecular dynamics (MD) simulations corroborate these findings (Figs. 3e, f, and S10). The n(r) value is 0.21, which is ascribed to the interaction between Li^+^ and DME/DOL. Notably, the intensity of Li−O peaks in the P/Ce-NC system is significantly diminished versus the blank electrolyte, signaling a reconstructed Li^+^ solvation environment. Quantitative analysis reveals that the introduction of P/Ce-NC reduces the coordination number (CN) of Li^+^−DME (from 4.9 to 4.4) and Li^+^−DOL (from 0.16 to 0.15). This creates a labile coordination environment that reduces desolvation energy barriers for Li(DME) (0.3 eV) and Li(DOL) (0.39 eV) in the P/Ce-NC system compared to the commercial electrolyte system. This confirms that the optimized Ce 4*f* orbitals can effectively anchor solvated O atoms, thereby catalyzing the desolvation process.

To experimentally validate the influence of the P/Ce-NC and PNC catalyst layer on the solvation structure, *in situ* Raman spectroscopy was employed. During the discharge process, the spectra collected on the cathode side of the P/Ce-NC battery show a significant decrease in the signal of the Li−DME peak (~875 cm^−1^), confirming the weakened Li^+^-solvent interaction in the solvation sheath during the initial discharge stage (Figs. 3g and S11). Notably, this suppressed signal remains stable and even slightly weakens further near 1.7 V. This sustained suppression indicates that P/Ce-NC enables a long-lasting weak interaction between Li^+^ and solvent molecules. In stark contrast, the intensity of the Li−DME peak in the PNC battery remains pronounced throughout the discharge process, indicating strong Li^+^-solvent interaction (Fig. 3h). This analysis directly elucidates that the P/Ce-NC catalyst layer effectively modulates the solvation environment, favoring the Li^+^-desolvation process.

To further elucidate the role of the P/Ce-NC catalyst layer in regulating the Li^+^ solvation structure, Raman spectroscopy was conducted on the cathode-side electrolyte of cycled LSBs (Fig. 3j). It can be observed that in the presence of the P/Ce-NC catalyst layer, the proportions of SSIP, CIP, and AGG are 50.19%, 29.89%, and 19.92%, respectively. In contrast, for the electrolyte with the PNC catalyst layer, the corresponding percentages are 67.63% (SSIP), 23.52% (CIP), and 8.84% (AGG). This shift occurs because the weakened Li^+^-solvent interaction facilitates anion incorporation into the primary solvation sheath, thereby promoting the formation of CIP and AGG structures. Collectively, these results confirm that the P/Ce-NC catalyst layer effectively induces the reorganization of the Li^+^ solvation structure, lowers the desolvation energy barrier, and accelerates the Li^+^ desolvation process.

The Li^+^ transference number (${\mathrm{t}}_{{Li}^{+}}$) and ionic conductivity (σ) reflect the efficiency of carrier diffusion and ionic mobility, which in turn influence the dissociation efficiency of the solvation sheath. The measured ${\mathrm{t}}_{{Li}^{+}}$ in the P/Ce-NC catalyst layer is 0.45, which is much higher than that of the PNC (0.11) (Fig. S13a and b). The observed enhancement in ${\mathrm{t}}_{{Li}^{+}}$ is attributed to an optimized solvation structure within the electrolyte. Similarly, Fig. S13c presents the σ values of P/Ce-NC and PNC catalyst layer, which are 0.33 and 0.24 mS cm^−1^, respectively. This further confirms the crucial role of P/Ce-NC in accelerating the desolvation process.

### Achieving a Stable Lithium Anode/Electrolyte Interface by Accelerated Li^+^-Solvents Desolvation Process

**Fig. 4:**
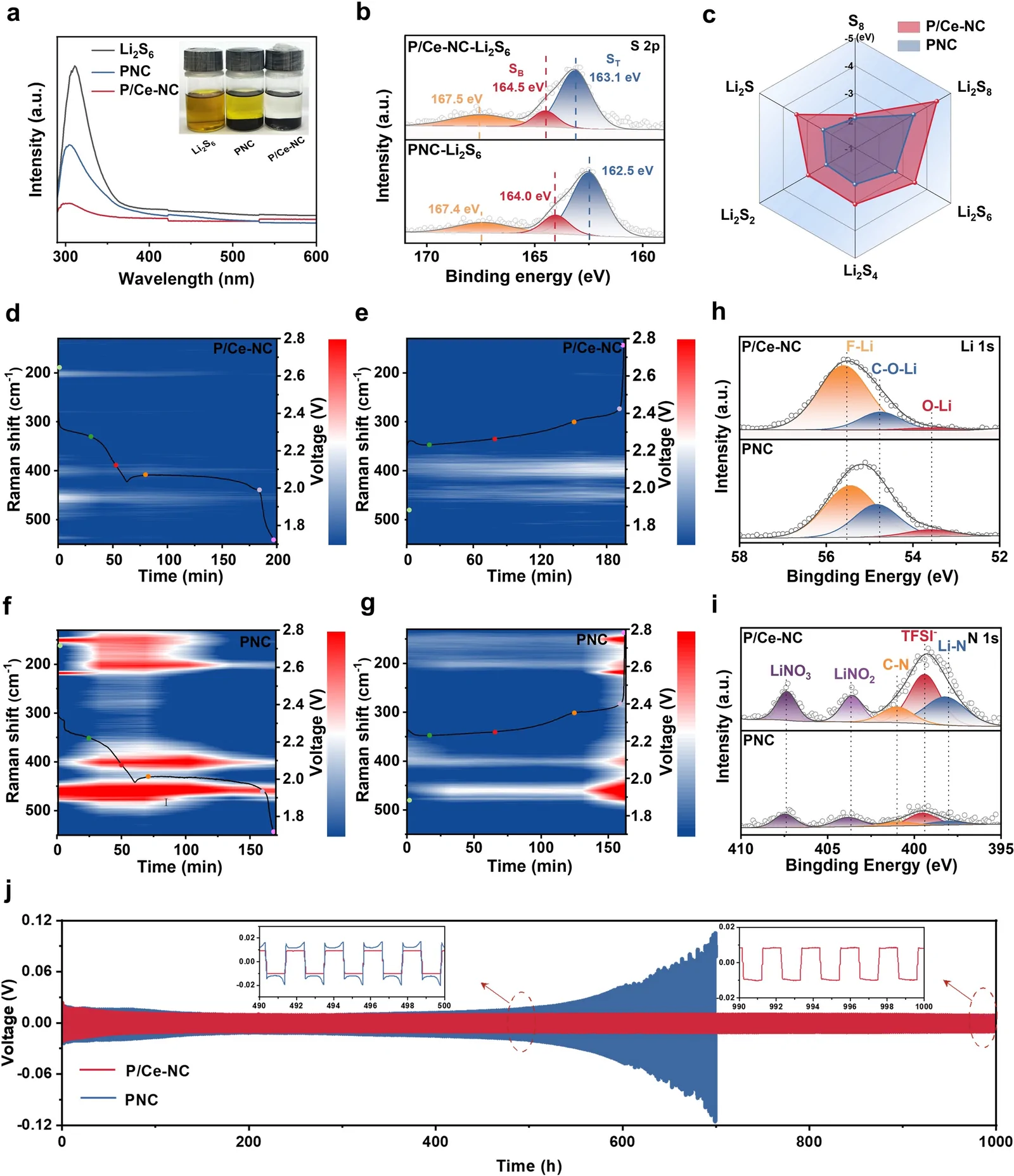
**a** UV–vis adsorption spectroscopy of Li_2_S_6_ solution with P/Ce-NC and PNC (the illustration is a digital photo of the Li_2_S_6_ adsorption experiment). **b** XPS spectra of S 2*p* in the P/Ce-NC and PNC after absorbing Li_2_S_6_ solution. **c** Adsorption energy of Li_2_S_n_ on different catalysts. *In situ* Raman spectra of **d, e** P/Ce-NC and **f, g** PNC. **h** Li 1*s*, and **i** N 1*s* XPS spectra of the lithium anode after 50 cycles of the battery with P/Ce-NC catalyst layer. **j** Long cycle performance of Li//Li symmetrical cells

Visual adsorption experiments were conducted to evaluate the adsorption capacity of LiPSs. As shown in Fig. 4a, the solution is rapidly decolored by the P/Ce-NC catalyst, confirming that the P/Ce-NC catalyst has a stronger adsorption effect on Li_2_S_6_. This observation was corroborated by UV-vis spectroscopy, where the peaks show a more intense decline after adsorption. As shown in Fig. 4b, XPS analysis was further tested to investigate the chemical interaction between P/Ce-NC and Li_2_S_6_. In the S 2*p* XPS spectrum of P/Ce-NC-Li_2_S_6_, both the peaks of terminal sulfur (S_T_, 163.1 eV) and bridged sulfur (S_B_,164.5 eV) shift toward higher binding energy than PNC-Li_2_S_6_ [42, 43]. This change indicates a strong interaction between P/Ce-NC and Li_2_S_6_.

The optimized configuration and adsorption energies of different substrates toward LiPSs were shown and calculated in Figs. 4c, S15, and S16. The higher adsorption energies of LiPSs (S_8_, Li_2_S_8_, Li_2_S_6_, Li_2_S_4_, Li_2_S_2_, Li_2_S) on P/Ce-NC indicate strong adsorption capacity. An in-depth analysis of the Ce–S hybridization mechanism was conducted using DOS. As shown in Fig. S14, it can be observed that the incorporation of phosphorus enhances the bonding orbital occupation of the Ce orbital, thereby strengthening *f-d-p* hybridization. We further evaluated the precipitation behavior of Li_2_S (Fig. S17). The battery with P/Ce-NC catalyst layer exhibits a higher Li_2_S deposition capacity (192.43 mAh g^−1^), indicating accelerated conversion kinetics. In addition, the CV curves of the symmetric cells with P/Ce-NC catalyst layer also exhibit higher peak current responses, indicating high catalytic ability toward LiPSs conversion (Fig. S18).

*In situ* Raman spectroscopy on the anode side directly evaluates shuttle inhibition (Figs. 4d–g and S19). In the Raman spectrum of the PNC, a prominent characteristic peak of long-chain S_8_^2−^ (150, 218, and 477 cm^−1^) and short-chain S_6_^2−^/S_4_^2−^ (400 cm^−1^) and S_4_^2−^/S_3_^2−^ (460 cm^−1^) can be detected. As the discharge progresses, the peak intensity of S_8_^2−^ gradually weakens, while the peaks of short-chain LiPSs gradually strengthen. As the charging proceeds, the peak of S_8_^2−^ reaches its maximum. These results prove that the PNC system exhibits a severe shuttle effect and a self-discharge phenomenon. In contrast, the consistently weak characteristic signals of LiPSs during the charge-discharge process in the P/Ce-NC system are due to effective suppression of the shuttle effect. Optical images of the Li anode after cycling in an *in situ* Raman sample chamber are also shown in Fig. S20, with more LiPSs observed in the PNC system. This further confirms the inhibitory effect of the P/Ce-NC catalyst layer on LiPSs.

The structure and chemical composition of the solid electrolyte interphase (SEI) are crucial indicators for evaluating the stability of the lithium anode/electrolyte interface. XPS analysis was performed on cycled lithium anodes to explore the effect of the P/Ce-NC catalyst layer on the SEI. Inorganic-rich SEI components exhibit enhanced Li^+^ conductivity and high interfacial stability, effectively promoting uniform deposition of Li^+^. In contrast, organic components generally feature low ionic conductivity and poor thermal stability [44]. As shown in the F 1*s* XPS spectrum (Fig. S21), intense characteristic peaks of C−F (688.3 eV) and Li−F (685.1 eV) are observed for the P/Ce-NC system, confirming the accumulation of inorganic fluorides. The Li 1*s* XPS spectrum further reveals that the SEI in the P/Ce-NC system contained a mixture of inorganic (such as F−Li) and organic (such as C−O−Li and O−Li) components (Fig. 4h). Notably, the P/Ce-NC system shows significantly attenuated organic fraction compared to the PNC. Additionally, the N 1*s* XPS spectrum indicates a higher content of Li_3_N in the battery with the P/Ce-NC catalyst layer (Fig. 4i). It is not difficult to deduce that the P/Ce-NC catalyst layer plays a decisive role in suppressing anode side reactions and regulating lithium deposition behavior.

Galvanostatic discharge-charge test using Li//Li symmetric cells was employed to characterize the improvement of different catalyst layers on the lithium plating/stripping cycling ability (Fig. 4j). The Li//Li symmetric cell with the P/Ce-NC catalyst layer exhibits the minimum overpotential (0.01 V) at 0.5 mA cm^−2^ and achieves a prolonged cycling lifespan exceeding 1000 h. In contrast, the overpotential of the cell with the PNC catalyst layer rapidly increases to 0.12 V at 700 h. The excellent cycling performance arises from the P/Ce-NC-induced solvation regulation, which ensures uniform Li⁺ deposition and a stable lithium anode interface.

### Evaluation of Redox Reaction Kinetics

**Fig. 5:**
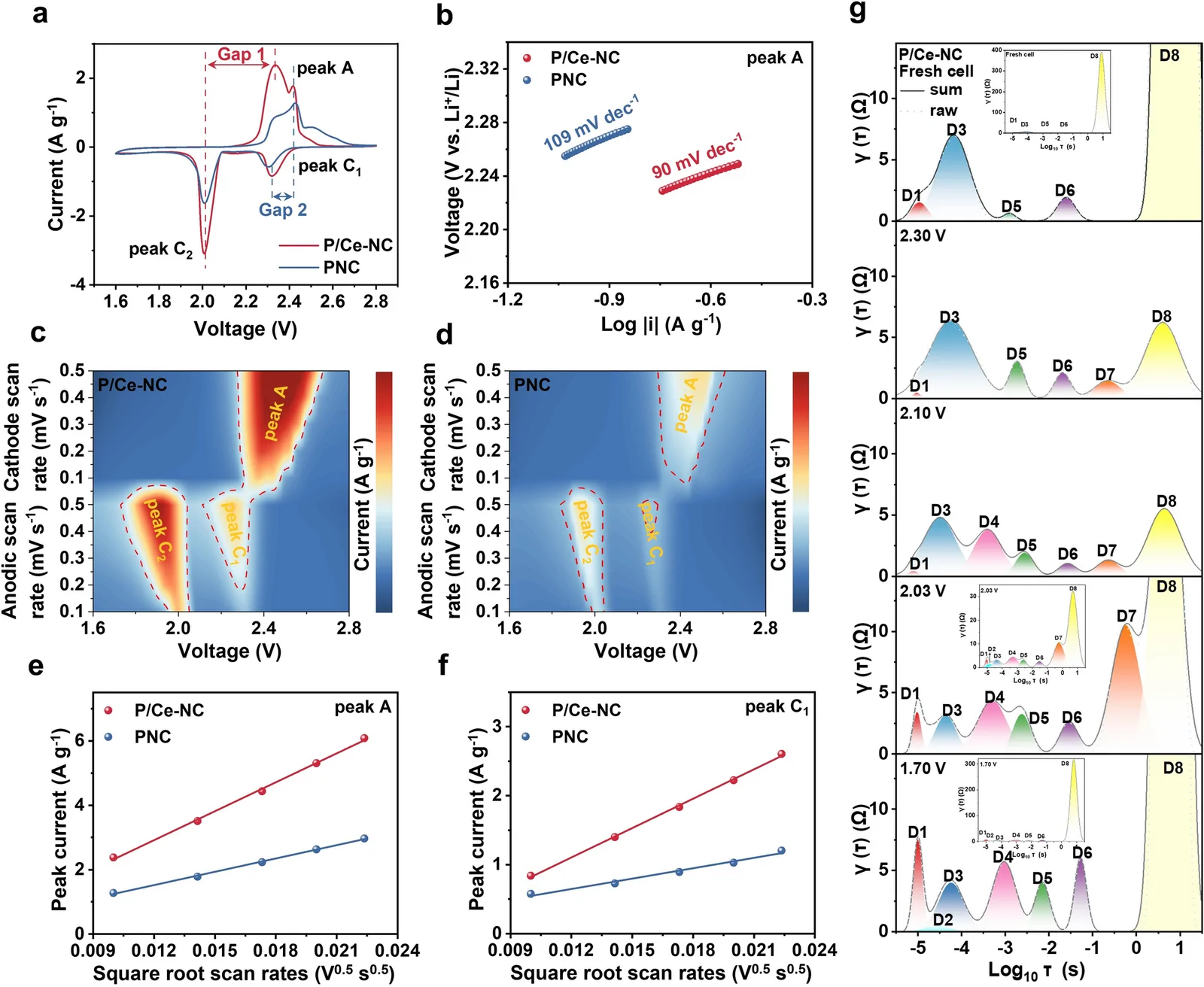
**a** CV curves, and **b** Tafel plots based on the peak A. CV curves of the battery with **c** P/Ce-NC and **d** PNC catalyst layer at various voltage scan rates. The peak current versus the square root of scan rate at peak **e** A, **f** C_1_. **g** DRT profiles of the battery with P/Ce-NC catalyst layer at different voltages

The cyclic voltammetry (CV) curves of the battery with P/Ce-NC catalyst layer show two sharper oxidation peaks (the reverse reaction of Li_2_S_2_/Li_2_S being oxidized to S_8_), revealing high catalytic activity and rapid multiphase conversion kinetics (Fig. 5a). In addition, the battery with the P/Ce-NC catalyst layer exhibits a smaller oxidative peak potential gap (Gap 1, 0.32 V) and a smaller reductive peak potential gap (Gap 2, 0.10 V) than PNC, elucidating the faster redox kinetics. The further obtained Tafel slopes through CV curve are shown in Figs. 5b and S22. The battery with the P/Ce-NC catalyst layer displays a smaller slope (90 mV dec^−1^) than the PNC (109 mV dec^−1^), indicating excellent electrochemical activity and redox kinetics of the P/Ce-NC catalyst layer. Moreover, the CV curves at different scan rates show smaller polarization potentials and larger peak currents in the battery with the P/Ce-NC catalyst layer, corresponding to faster Li^+^ migration and sulfur conversion (Fig. 5c, d). Figure 5e, f and S23 exhibits linear fitting of peak currents versus the square root scan rate of the batteries with different catalyst layers. As shown in Table S5, the mass transfer kinetics were quantified using the Li^+^ diffusion coefficient (${\mathrm{D}}_{{Li}^{+}}$). The battery with P/Ce-NC catalyst layer exhibits larger ${\mathrm{D}}_{{Li}^{+}}$, which is attributed to the accelerated desolvation process.

To reveal the electron transfer and ion diffusion mechanisms, ex situ EIS measurements were performed at different potentials (Figs. S24 and S25). The distribution of relaxation times (DRT) curve in Figs. 5g and S26 shows eight peaks (D1-D8), D1, D2, D3, and D4 peaks at 10.14, 13.39, 44.06, and 504.70 μs correspond to the polarization of the high-frequency semicircle; the D5 and D6 peaks at 2.45 and 2.95 ms are considered to be reactions determined in the mid-frequency semicircle; the D7 and D8 peaks at 0.46 and 10.01 s constitute the diffusion process in the low-frequency region[45]. In detail, D1-D8 represent interparticle transmission resistance, relaxation related to double-layer capacitance, and SEI film resistance, charge transfer of the positive electrode, charge transfer reactions, charge transfer reactions, polysulfide diffusion, and diffusion. The DRT curve begins to change significantly after the start of discharge (Fig. S27, Tables S6 and S7). As shown in the battery with P/Ce-NC catalyst layer, sulfur is reduced to long-chain Li_2_S_8_/Li_2_S_6_, and the electrode loses its blocking behavior due to the transformation of the sulfur electrode. This change is reflected in the ion diffusion resistance to (the D8 peak changes from 219.10 Ω in the initial stage to 6.14 Ω at 2.30 V). The Li_2_S_2_/Li_2_S layer gradually forms as the discharge progresses to 2.03 V, resulting in a significant increase in D7 and D8 peaks. Furthermore, most peaks reach their maximum resistance at 1.70 V, which suggests the majority of LiPSs are being reduced to Li_2_S. The disappearance of the D7 peak at this stage correlates with the role of the P/Ce-NC catalyst layer in suppressing the shuttle effect of LiPSs [46].

### Electrochemical Performance of LSBs

**Fig. 6:**
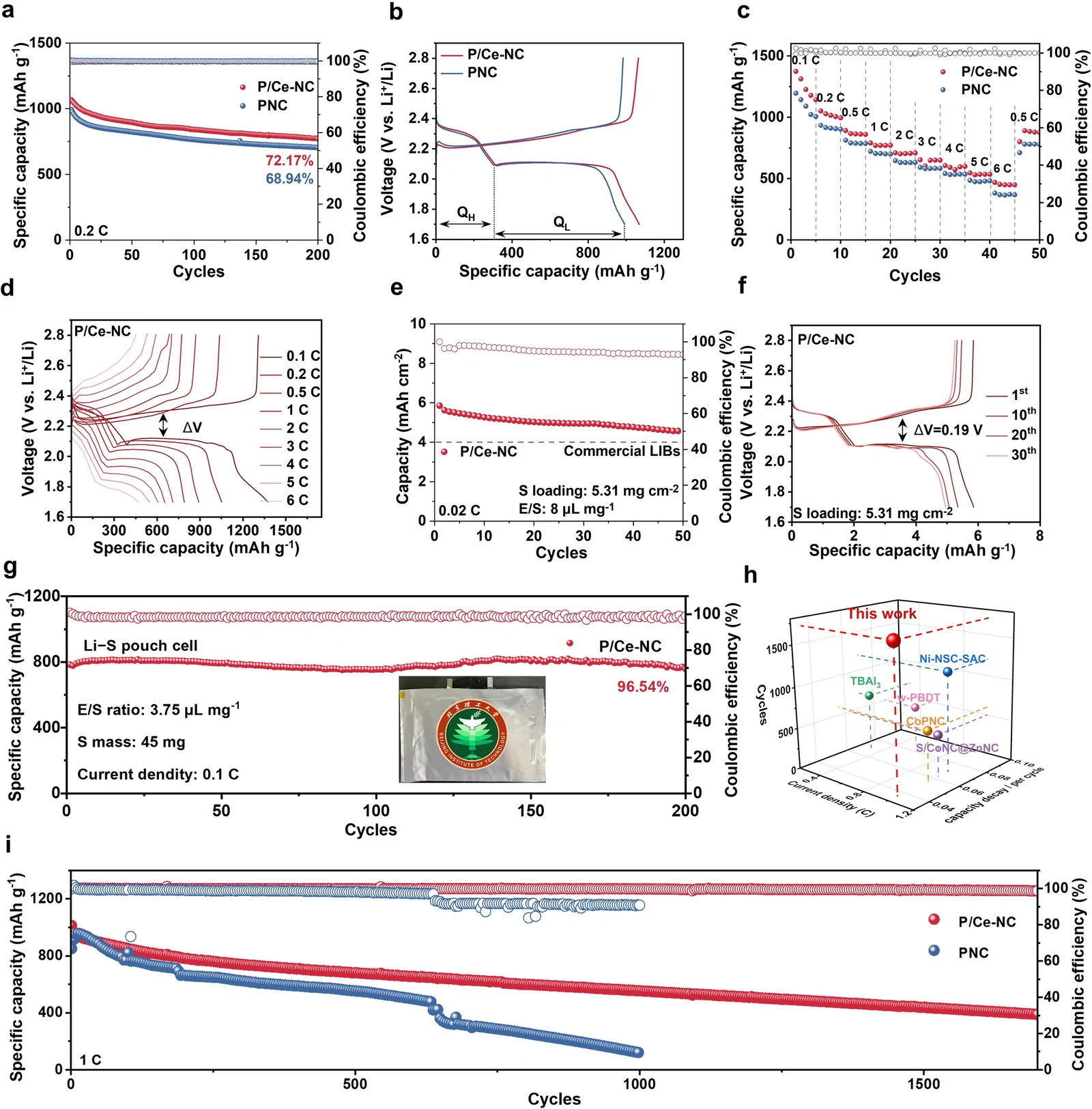
**a** Cycling performance, **b** charge/discharge profiles, and **c** rate performance of the battery with P/Ce-NC and PNC catalyst layer. **d** Charge-discharge profiles of the battery with P/Ce-NC catalyst layer from 0.1 C to 6 C. **e** Cycle performance at 0.1 C with high sulfur loading, **f** charge-discharge profiles of the battery with P/Ce-NC catalyst layer. **g** Cycling performance of the pouch cell with P/Ce-NC catalyst layer. **h** Performance comparison of P/Ce-NC and reported LSBs based on solvation modulation and SACs. **i** Long-term cycling performance at 1 C

To evaluate the impact of the electrochemical performance of the battery with different catalyst layers, a cycling test was conducted (Fig. 6a). The battery with the P/Ce-NC catalyst layer exhibits an initial discharge capacity of 1134.18 mAh g^−1^ and retains 815.46 mAh g^−1^ at 0.2 C after 200 cycles, corresponding to a 72.17% retention, with a stable coulombic efficiency of 99.65%. To effectively assess the excellent polysulfides conversion performance of the P/Ce-NC catalyst layer, the high-voltage plateau capacities (Q_H_) and low-voltage plateau capacities (Q_L_) were measured. As shown in Fig. 6b, the Q_L_/Q_H_ of the battery with the P/Ce-NC catalyst layer (2.46) is higher than that of PNC (2.23), confirming high sulfur utilization. Subsequently, the rate capability of the battery was tested in Fig. 6c. The battery with P/Ce-NC catalyst layer stands out with the capacities of 1374.31, 1051.60, 891.46, 790.53, 710.27, 653.39, 606.83, 546.76, 469.09, and 800.75 mAh g^−1^ at rates of 0.1, 0.2, 0.5, 1, 2, 3, 4, 5, 6, and 0.5 C. The battery with the PNC catalyst layer exhibits low discharge specific capacity at various rates, revealing high catalytic activity of P/Ce-NC. The charge-discharge profiles at different rates are shown in Figs. 6d, S28, and S29. The voltage gap of P/Ce-NC between the charge and discharge plateaus is significantly lower than that of PNC at different rates.

In addition, the battery with the P/Ce-NC catalyst layer exhibits a superior reversible areal capacity of 5.85 mAh cm^−2^ with a high sulfur loading of 5.31 mg cm^−2^ and a low E/S of 8 μL mg^−1^ (Fig. 6e). The charge-discharge curves corresponding to various cycles exhibit a typical and complete dual plateau. The voltage gap between the charge and discharge plateaus is low (0.19 V), highlighting its potential in practical applications (Fig. 6f). The pouch cell with P/Ce-NC catalyst layer exhibits an initial specific capacity of 784.55 mAh g^−1^ at 0.1 C, maintains 96.54% of its capacity after 200 cycles (Fig. 6g). Furthermore, in terms of capacity retention, the battery with P/Ce-NC catalyst layer also displays a high specific capacity of 939.10 mAh g^−1^ at 1 C, corresponding to a decay rate of 0.036% per cycle over 1700 cycles (Fig. 6i). This performance is markedly superior to that of the battery with the PNC catalyst layer, which exhibits a higher capacity decay of 0.058% per cycle over 1000 cycles. The long-cycle performance of LSBs based on SACs and solvation modulation is compared in Fig. 6h and Table S8, which demonstrates the excellent electrochemical performance of the battery with the P/Ce-NC catalyst layer in this work.

## Conclusion

In summary, we propose an innovative catalyst desolvation strategy to reduce the desolvation energy barrier and enhance the sulfur redox kinetics. The introduced phosphorus into the second coordination sphere of the Ce–N_4_ site enhances the electron occupancy of the* f*_*y3x*_^*2*^ and* f*_*z*_^*3*^ orbitals of Ce, which weakens the interaction between Li^+^ and solvent molecules. Therefore, the Li^+^ solvation structure shifts toward an anion-dominated configuration rich in AGG and CIP, effectively lowering the Li^+^-solvent desolvation energy barrier. In addition, the strengthened *f-d-p* orbital hybridization effectively inhibits the diffusion of LiPSs through the interlayer, thereby alleviating parasitic reactions on the lithium metal anode. As a result, the LSBs with P/Ce-NC catalyst layer deliver a low decay rate of 0.036% per cycle over 1700 cycles at 1 C. This catalyst desolvation strategy, coupling Li^+^ desolvation and enhanced sulfur reaction kinetics, offers a promising avenue for the development of long lifespan LSBs.

## Supplementary Information

Below is the link to the electronic supplementary material.

Supplementary file1 (DOCX 12996 KB)
