# Supplementary material for: Breaking the Limitations of Sulfur Redox Kinetics by Accelerated Li+-Desolvation in Lithium–Sulfur Batteries
Source: Nanomicro Lett. 2026 May 27;18:388. doi: 10.1007/s40820-026-02232-6 (PMC13216370; doi:10.1007/s40820-026-02232-6)
Supplement: Supplementary file 1 — Supplementary file1 (DOCX 12996 KB) [file 40820_2026_2232_MOESM1_ESM.docx]

Supporting Information for

Breaking the Limitations of Sulfur Redox Kinetics by Accelerated Li^+^-Desolvation in Lithium–Sulfur Batteries

Tan Wang^1^, Zhenhua Wang^1,^ *, Xiaotian Gao^1^, Zhe Bai^1^, Wanning Liu^1^, Yu Bai^1^, David Rooney^2,^ *, Kening Sun^1,^ *

^1^Beijing Key Laboratory of Green Hydrogen and Fuel Cells, School of Chemistry and Chemical Engineering, Beijing Institute of Technology, Beijing 100081, P. R. China

^2^School of Chemistry and Chemical Engineering, Queen's University Belfast, Belfast, Northern Ireland, BT9 5AG, UK

*Corresponding authors. E-mail: [wangzh@bit.edu.cn](mailto:wangzh@bit.edu.cn) (Zhenhua Wang); [d.rooney@qub.ac.uk](mailto:d.rooney@qub.ac.uk) (David Rooney); [sunkn@bit.edu.cn](mailto:sunkn@bit.edu.cn) (Kening Sun)

**S1 Materials characterizations**

XRD was performed to determine the composition of the samples (Ultima IV-185, Cu Kα radiation). The Raman spectra were measured with a 532 nm laser (HORIBA, HR Evolution). The morphology was obtained by SEM (Hitachi S-8230), TEM, and HR-TEM (JEM-2100F). The XPS spectra were measured to test the surface element characteristics (Kratos Axis Supra). X-ray absorption data were collected at the Aichi Synchrotron Radiation Center. ICP-OES was used to test the content of Ce and P. HAADF-STEM was collected using Theims Z field emission electron microscope (FEI, Netherlands).

**S2 Electrochemical measurements**

We prepared the cathode slurry through mixing CNT/S (CNT and sulfur powders were mixed in a 7:3 ratio and then heated at 155°C for 12 h), super P, and PVDF in a mass ratio of 7:2:1. With a mass ratio of 3:6:1, the modified interlayer slurry was prepared from P/Ce-NC (or PNC or P/Ce@NC), CNT, and PVDF. The cathode slurry and modified interlayer slurry were all stirred for 12 h and then loaded onto the aluminum foil and Celgard 2500 separator. Under excessive-electrolyte conditions (electrolyte-to-sulfur (E/S) ratio is 30 μL/mg(S)), the sulfur mass loading was 1.0-1.3 mg cm^−2^. While under lean-electrolyte conditions (E/S ratio is 8 μL/mg(S)), the sulfur mass loading was set at 5.31 mg cm^−2^. Then kept at 60℃ overnight. LSBs used Li metal as the anode (φ14 mm), CNT/S as the cathode (φ12 mm), and P/Ce-NC or P/Ce@NC or PNC as the separator (φ19 mm), 1 M dilithium (trifluoromethane sulfonylimide) imide (LiTFSI) in DOL/DME (1:1 volume ratio) with 1 wt% % LiNO_3_ as the electrolyte (Canrd Technology Co. Ltd). The Li–S pouch cell was assembled using a sulfur cathode (45 mg sulfur loading) and a lithium foil anode (100 μm thick), with dimensions of 3×4 cm^2^ and 4×5 cm^2^, respectively. The E/S ratio was kept at 3.75 μL/mg(S). Galvanostatic charge/discharge cycling between 1.7 and 2.8 V was tested by a Neware battery testing system. The pouch cells need to be tested using battery clamps (Changgao New Materials Co., Ltd). The in-situ Raman measurements were conducted on custom-assembled LSBs using a sample chamber with quartz optical windows. Raman spectra were acquired simultaneously with the discharge process from the open-circuit voltage to 1.7 V at a rate of 0.15 C. The CHI660e (Shanghai Chenhua) electrochemical workstation was used for CV, symmetric CV, and EIS. Assembly of Li_2_S_6_ symmetrical cell: The working electrodes were fabricated by creating a homogeneous dispersion of 20 mg catalyst (P/Ce-NC or PNC) in 2 mL ethanol. This solution was uniformly applied onto a carbon paper substrate until an areal loading of 0.5 mg cm^−2^ was obtained. Symmetrical cells were constructed containing 40 μL of Li_2_S_6_ solution sandwiched between two identical electrodes and a Celgard 2500 separator. Symmetric CV tests were conducted on the Li_2_S_6_ symmetric cell within the voltage range of -1.0 V to 1.0 V. In addition, CV tests were conducted on Li–S batteries within the voltage range of 1.6-2.8V.

**S3 Li_2_S nucleation test**

The cell was assembled with a carbon paper-based working electrode (loaded with P/Ce-NC or PNC), a lithium foil counter electrode, a Celgard 2500 separator, and the electrolyte of Li_2_S_8_ solution of 2.0 mol L^-1^ and a blank electrolyte, respectively. We subjected the batteries to galvanostatic discharge to 2.1 V (0.112 mA) and then potentiostatic discharge at 2.09 V until the current fell below 10 μA.

**S4 Theoretical calculations**

We employed the Vienna Ab initio Simulation Package (VASP) to carry out spin-polarized density functional theory (DFT) calculations [S1]. The exchange-correlation energy was treated with the Perdew-Burke-Ernzerhof (PBE) functional [S2]. The cut-off energy was set to 520 eV, and a vacuum layer was set at least 15 Å. In addition, the spin polarization was considered in the DFT calculations. The electronic energy convergence was set at 10⁻^4^ eV [S3]. The adsorption energy (Eads) was defined as:

$$E_{binding}=E_{total}-E_{slab}-E_{ads} (S1)$$

$E_{total}$, $E_{slab}$ and $E_{ads}$ are the energy of total, the energy of optimized structure, and the energy of LiPSs. The Gibbs free energy was calculated using the following formula.

$G=G_{correction}+E_{DFT}=E_{ZPE}+\Delta U-TS+E_{DFT}=U-TS+E_{DFT}=G\left( T \right)+E_{DFT}$ (S2)

Where $G_{correction}$ is the corrected Gibbs free energy by zero-point energy, $E_{DFT}$ denotes the energy obtained by DFT calculation, $U, S$ and $T$ represent the internal energy, the entropy, and the absolute temperature, respectively. $E_{ZPE}$ represents the zero-point energy. $G\left( T \right)$ can be calculated by the VASPKIT code after the frequency calculation of adsorbed LiPS.

**S5 Supplementary Figures and Tables**

**
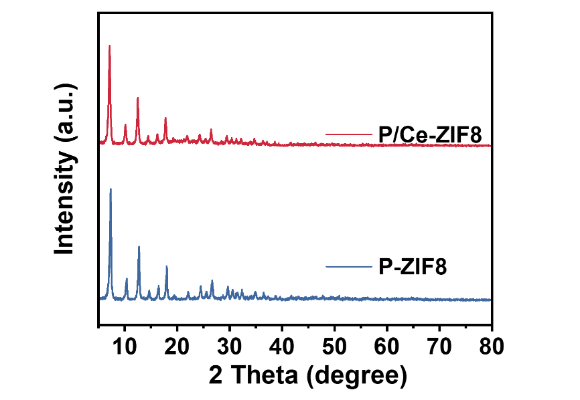
**

**Fig. S1** XRD patterns of P/Ce-ZIF8 and P-ZIF8.

**
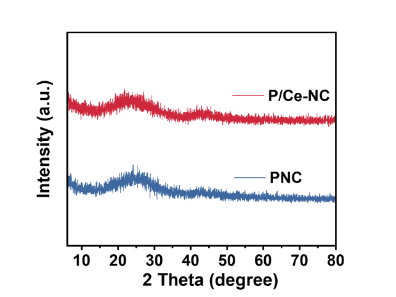
**

**Fig. S2** XRD patterns of P/Ce-NC and PNC.

**
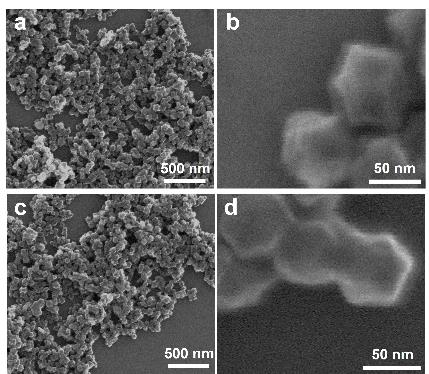
**

**Fig. S3** SEM images of (**a, b**) P/Ce-NC and (**c, d**) PNC.

**
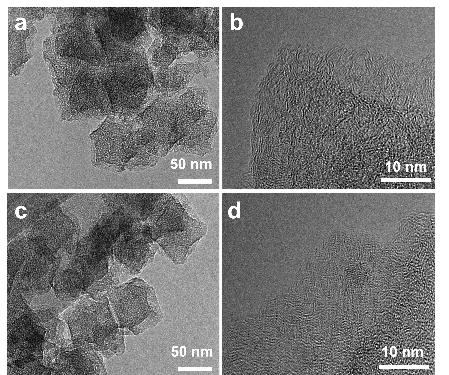
**

**Fig. S4** TEM and HRTEM of (**a, b**) P/Ce-NC and (**c, d**) PNC.


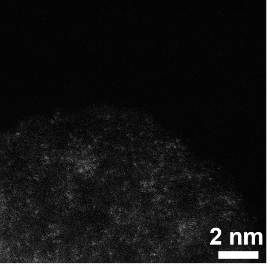


**Fig. S5** HAADF image of the P/Ce-NC.

**Fig. S6** BET test of P/Ce-NC.

**Fig. S7** XPS spectra of N 1s in the P/Ce-NC.

**Fig. S8** PDOS of the Ce *4f* orbitals in Ce-NC.

**Fig. S9** PDOS of the Ce *3d* orbitals in (**a**) P/Ce-NC and (**b**) Ce-NC.

**Fig. S10** The snapshots of commercial electrolyte (**a**) without and (**b**) with P/Ce-NC via MD simulation.

**Fig. S11** *In-situ* Raman spectra with (**a**) P/Ce-NC and (**b**) PNC catalyst layer.

**
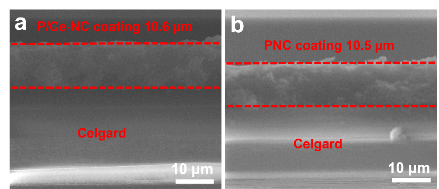
**

**Fig. S12** SEM images of (**a**) P/Ce-NC and (**b**) PNC catalyst layer.

**
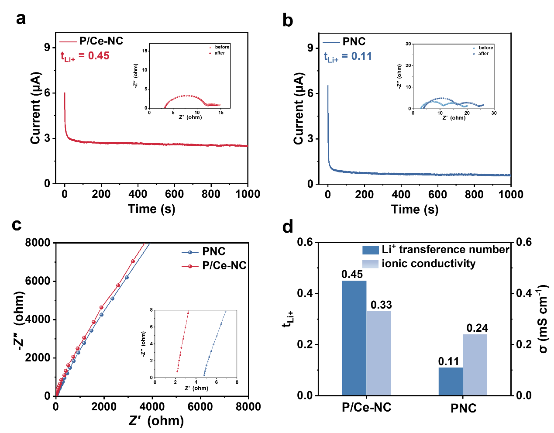
**

**Fig. S13** The amperometric *i*-*t* and EIS curves of the battery with (**a**) P/Ce-NC and (**b**) PNC interlayer. (**c**) Ionic conductivity. (**d**) Li^+^ transference number and the ionic conductivity of P/Ce-NC and PNC.

**Fig. S14** PDOS of P/Ce-NC-Li_2_S_2_ and Ce-NC-Li_2_S_2_.

**Fig. S15** Geometric configurations of (**a**) S_8_, (**b**) Li_2_S_8_, (**c**) Li_2_S_6_, (**d**) Li_2_S_4_, (**e**) Li_2_S_2,_ and (**f**) Li_2_S on P/Ce-NC.

**Fig. S16** Geometric configurations of (**a**) S_8_, (**b**) Li_2_S_8_, (**c**) Li_2_S_6_, (**d**) Li_2_S_4_, (**e**) Li_2_S_2,_ and (**f**) Li_2_S on PNC.

**Fig. S17** Li_2_S nucleation test for the battery with (**a**) P/Ce-NC and (**b**) PNC catalyst layer.


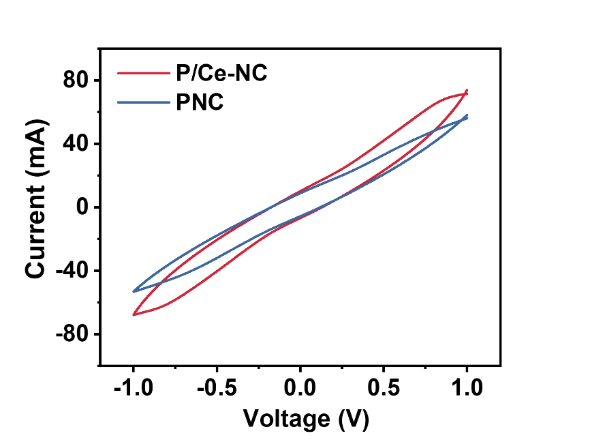


**Fig. S18** CV curves of symmetric cells with Li_2_S_6_.

**Fig. S19** *In-situ* Raman spectra of (**a, b**) P/Ce-NC and (**c, d**) PNC system.

**
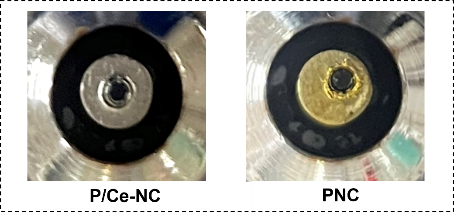
**

**Fig. S20** Optical images of the Li anode after cycling in an *in situ* Raman sample chamber.

**Fig. S21** F 1s XPS spectra of the lithium anode after 50 cycles of the battery with P/Ce-NC catalyst layer.

**Fig. S22** Tafel plots based on the peak C_1_.

**Fig. S23** The peak current versus the square root of scan rate at peak C_2_.

**Fig. S24** EIS measurements of P/Ce-NC-based battery, (**a**) fresh cell, (**b**) discharge to 2.30 V, (**c**) discharge to 2.10 V, (**d**) discharge to 2.03 V, and (**e**) discharge to 1.70 V.

**Fig. S25** EIS measurements of PNC-based battery, (**a**) fresh cell, (**b**) discharge to 2.30 V, (**c**) discharge to 2.10 V, (**d**) discharge to 2.02 V, and (**e**) discharge to 1.70 V.

**Fig. S26** DRT profiles of PNC-based battery at different voltages (**a**) fresh, (**b**) discharge to 2.30 V, (**c**) discharge to 2.10 V, (**d**) discharge to 2.02 V, and (**e**) discharge to 1.70 V.

**Fig. S27** Polarization resistance of D1-8 for P/Ce-NC-based battery at different voltages.

**Fig. S28** The charge-discharge profiles of the PNC-based battery from 0.1 C to 6 C.

**Fig. S29** The corresponding voltage gap value of Li−S batteries using different catalyst layers.

**Fig. S30** EIS performance of battery with P/Ce-NC catalyst layer (**a**) before and (**b**) after 50 cycles.

**Discussion:** The P/Ce-NC battery exhibits significantly lower resistance than the PNC battery, both before and after cycling. This indicates that the P/Ce-NC catalyst layer could promote the conversion kinetics of LiPSs during long cycles.

**
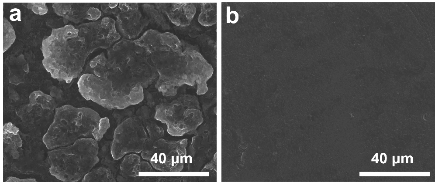
**

**Fig. S31** SEM images of the lithium anode after cycling of (**a**) PNC and (**b**) P/Ce-NC-based battery.

**Discussion:** The anode after cycling was tested by SEM, and it is evident that the PNC battery exhibits severe corrosion. In stark contrast, the lithium anode of the P/Ce-NC battery exhibits a uniform surface. This finding further validates that the P/Ce-NC can effectively suppress the shuttle effect and promote uniform deposition of the lithium anode.


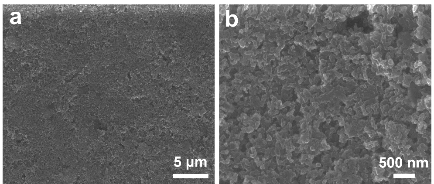


**Fig. S32** SEM images of the P/Ce-NC catalyst layer after 1000 cycles of the P/Ce-NC-based battery.

**Discussion:** As shown in Fig. S32, after 1000 cycles, the morphology of the P/Ce-NC catalyst remained highly intact, and no structural collapse was observed. This observation further confirms the excellent structural stability of the P/Ce-NC catalyst.

**Fig. S33** XRD patterns of (**a**) P/Ce@NC and (**b**) P/Ce@ZIF8.

**Discussion:** The reference sample of P/Ce@NC was synthesized by an identical method with a high loading of Ce. The XRD pattern showed identical diffraction patterns, and in addition, the diffraction peak corresponding to CeP appears.

**
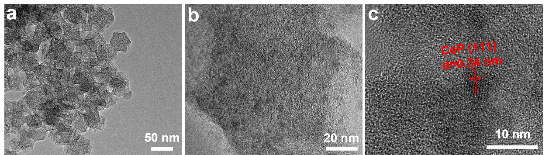
**

**Fig. S34** (**a, b**) TEM and (**c**) HRTEM image of P/Ce@NC.

**Discussion:** The TEM image clearly shows the shape of a rhombic dodecahedron and a large number of loosely arranged nanoparticles on its surface (Fig. S34b). The HRTEM image of P/Ce@NC exhibits clear lattice fringes (0.34 nm), corresponding to a single-layer CeP about (111) lattice plane.

**Fig. S35** (**a**) The rate performance and (**b**) cycling performance at 1 C of the battery with P/Ce@NC interlayer.

**Discussion:** Galvanostatic charge/discharge test was performed at different current densities, the discharge capacity is 1200.66, 934.36, 812.47, 721.78, 646.06, 591.94, 541.60, 486.23, 382.47 mAh g^-1^ of 0.1, 0.2, 0.5, 1, 2, 3, 4, 5, 6 C, respectively. The cyclic tests show the stability of P/Ce@NC-based battery under 1 C (Fig. S35b), which shows a reversible specific capacity of 937.75 mAh g^-1^ and a low average capacity fading of 0.058% per cycle.

**Table S1** Content of Ce elements in different catalysts

| sample | Mass  (g) | Constant volume  *V*_0_（mL) | Test element | Test the concentration of liquid elements  *C*_o_ (mg L^–1^) | Dilution factor  *f* | Concentration of the original solution  *C*_1_ (mg L^–1^) | Element content  *C*_x_ (mg kg^–1^) | Element content  *W* (%) |
| --- | --- | --- | --- | --- | --- | --- | --- | --- |
| Ce-129.0 | 0.0517 | 25 | Ce | 0.671 | 1 | 0.671 | 324.4 | 0.03% |
| Ce-322.5 | 0.0531 | 25 | Ce | 0.567 | 1 | 0.567 | 266.8 | 0.03% |
| Ce-645.0 | 0.0490 | 25 | Ce | 0.746 | 1 | 0.746 | 380.8 | 0.04% |

**Discussion:** As shown in Table S1, even when we significantly increased the amount of the Ce(NO_3_)_3_·6H_2_O precursor from 64.5 mg up to 129.0, 322.5, and even 645.0 mg (denoted as Ce-129.0, Ce-322.5, and Ce-645.0), no Ce species could be detected in the final products.

**Table S2** ICP-OES analysis result of the P/Ce-NC and P/Ce@NC

| Sample | Ce (wt.%) | | P (wt.%) | Zn (wt.%) |
| --- | --- | --- | --- | --- |
| P/Ce-NC | 5.30 | 2.80 | | 0.30% |
| P/Ce@NC | 10.53 | 3.12 | |  |

**Discussion:** As shown in Table S2, residual Zn content in P/Ce-NC is quantified to be as low as 0.30 wt. %, which would exert negligible influence on catalytic performance [S4, S5].

**Table S3** EXAFS fitting parameters at the Ce *L3*-edge for various samples

| Sample | Shell | *CN^a^* | *R*(Å)*^b^* | *σ*^2^(Å^2^)*^c^* | Δ*E*_0_(eV)*^d^* | *R* factor |
| --- | --- | --- | --- | --- | --- | --- |
| CeO_2_ | Ce-O | 2* | 2.138±0.001 | 0.0010±0.0001 | 5.9±0.4 | 0.0124 |
|  | Ce-O | 6* | 2.341±0.001 |  |  |  |
|  | Ce-O | 2* | 3.126±0.001 |  |  |  |
|  | Ce-Ce | 7* | 3.809±0.001 | 0.0005±0.0001 | 3.2±0.6 |  |
| P/Ce-NC | Ce-N | 3.9±0.6 | 2.505±0.001 | 0.0088±0.0001 | 1.8±0.9 | 0.0049 |
|  | Ce-O | 3.7±0.8 | 2.622±0.001 | 0.0160±0.0001 | 4.5±0.1 |  |

*^a^CN*, coordination number; *^b^R*, the distance to the neighboring atom; *^c^σ*^2^, the Mean Square Relative Displacement (MSRD); *^d^ΔE*_0_, inner potential correction; *R* factor indicates the goodness of the fit. *S*0^2^ was fixed to 0.730, according to the experimental EXAFS fit of CeO_2_ by fixing *CN* as the known crystallographic value. This value was fixed during EXAFS fitting, based on the known structure of CeO_2_. Fitting range: 3.0 ≤ *k* (/Å) ≤ 9.5 and 1.5 ≤ *R* (Å) ≤ 3.9 (CeO_2_); 2.0 ≤ *k* (/Å) ≤ 9.5 and 1.5 ≤ *R* (Å) ≤ 2.6 (P/Ce-NC). A reasonable range of EXAFS fitting parameters: 0.700 < *Ѕ*_0_^2^ < 1.000; *CN >* 0; *σ*^2^ > 0 Å^2^; |Δ*E*_0_| < 15 eV; *R* factor < 0.02.

**Table S4** The IpCOHP values of various samples

| Sample | Li−O_1#_ in DME | Li−O_2#_ in DME | | | Li−O in DOL |
| --- | --- | --- | --- | --- | --- |
| P/Ce-NC | 0.02 | | 0.12 | 0.06 | |
| DME | 0.13 | | 0.14 | 0.13 | |

**Table S5** The $\text{D}_{\mathrm{Li}^{+}}$ values for various electrochemical reaction processes

| Sample | Peak A | Peak C_1_ | Peak C_2_ |
| --- | --- | --- | --- |
| P/Ce-NC | 1.25×10^-7^ | 2.81×10^-8^ | 7.85×10^-8^ |
| PNC | 2.64×10^-8^ | 3.51×10^-9^ | 1.19×10^-8^ |

**Table S6** The resistance of the battery with P/Ce-NC layer at different voltages

| voltage | Fresh | 2.30 V | 2.10 V | 2.03 V | 1.70 V |
| --- | --- | --- | --- | --- | --- |
| D1/Ω | 0.72 | 0.09 | 0.11 | 0.81 | 2.01 |
| D2/Ω | 0 | 0 |  | 0.76 | 0.33 |
| D3/Ω | 6.45 | 7.93 | 4.50 | 1.88 | 2.91 |
| D4/Ω | 0.22 | 0 | 3.02 | 3.71 | 3.89 |
| D5/Ω | 0.10 | 1.25 | 0.95 | 1.69 | 1.72 |
| D6/Ω | 0 | 0.96 | 0.53 | 1.38 | 2.05 |
| D7/Ω | 0 | 1.08 | 0.88 | 8.77 | 0 |
| D8/Ω | 219.10 | 6.14 | 5.10 | 22.72 | 175.90 |

**Table S7** The resistance of the battery with PNC layer at different voltages

| voltage | Fresh | 2.30 V | 2.10 V | 2.03 V | 1.70 V |
| --- | --- | --- | --- | --- | --- |
| D1/Ω | 0.50 | 0.69 | 0.62 | 1.15 | 1.84 |
| D2/Ω | 0 | 0 | 0 | 0.17 | 0.52 |
| D3/Ω | 7.80 | 6.79 | 5.45 | 6.67 | 4.32 |
| D4/Ω | 5.01 | 2.72 | 2.26 | 3.15 | 2.67 |
| D5/Ω | 0.73 | 4.85 | 5.18 | 11.39 | 15.71 |
| D6/Ω | 0 | 1.23 | 0.69 | 3.26 | 3.68 |
| D7/Ω | 0 | 0.72 | 0.85 | 9.25 | 23.11 |
| D8/Ω | 303.17 | 6.48 | 7.99 | 40.70 | 100.04 |

**Table S8** Comparison of the cycling performance of P/Ce-NC and different reported LSBs based on single-atom catalysts and desolvation aspects

| Materials | Rate | Cycle Number | capacity decay/  per cycle | Reference |
| --- | --- | --- | --- | --- |
| S/CoNC@ZnNC DSNCs | 1 C | 500 | 0.0.63% | *Adv Mater*  *2024, 36, e2310547[S6]* |
| CoPNC | 1 C | 600 | 0.056% | *Adv. Funct. Mater.*  *2024, 2412279.[S7]* |
| Ni-NSC SAC | 1 C | 1200 | 0.070% | *Angew Chem Int Ed Engl*  *2024, e202418749.[S8]* |
| w-PBDT | 0.5 C | 500 | 0.088% | *Adv. Mater.*  *2024, 36, 2401473[S9]* |
| TBAl_3_ | 0.3 C | 700 | 0.07% | *Adv. Funct. Mater.*  *2025, 2500077[S10]* |
| P/Ce-NC | 1 C | 1700 | 0.036% | ***This Work*** |

**Supplementary References**

1. V. Wang, N. Xu, J.-C. Liu, G. Tang, W.-T. Geng, VASPKIT: a user-friendly interface facilitating high-throughput computing and analysis using VASP code. Comput. Phys. Commun. **267**, 108033 (2021). <https://doi.org/10.1016/j.cpc.2021.108033>
2. M. Chakraborty, P. Pal, B.R. Sekhar, Half metallicity in Pr_0.75_Sr_0.25_MnO3: a first principle study. Solid State Commun. **145**(4), 197–200 (2008). <https://doi.org/10.1016/j.ssc.2007.10.025>
3. C. Li, S. Qi, L. Zhu, Y. Zhao, R. Huang et al., Regulating polysulfide intermediates by ultrathin Co-Bi nanosheet electrocatalyst in lithium–sulfur batteries. Nano Today **40**, 101246 (2021). <https://doi.org/10.1016/j.nantod.2021.101246>
4. H. Shang, X. Zhou, J. Dong, A. Li, X. Zhao et al., Engineering unsymmetrically coordinated Cu-S(1)N(3) single atom sites with enhanced oxygen reduction activity. Nat. Commun. **11**(1), 3049 (2020). <https://doi.org/10.1038/s41467-020-16848-8>
5. Y. Xiong, J. Dong, Z.-Q. Huang, P. Xin, W. Chen et al., Single-atom Rh/N-doped carbon electrocatalyst for formic acid oxidation. Nat. Nanotechnol. **15**(5), 390–397 (2020). <https://doi.org/10.1038/s41565-020-0665-x>
6. L. Ren, K. Sun, Y. Wang, A. Kumar, J. Liu et al., Tandem catalysis inside double-shelled nanocages with separated and tunable atomic catalyst sites for high performance lithium-sulfur batteries. Adv. Mater. **36**(14), 2310547 (2024). <https://doi.org/10.1002/adma.202310547>
7. Y. Li, Z. Chen, X.-Y. Zhong, T. Mei, Z. Li et al., Modulating the coordination environment of co single-atom catalysts: impact on lithium-sulfur battery performance. Adv. Funct. Mater. **35**(2), 2412279 (2025). <https://doi.org/10.1002/adfm.202412279>
8. F. Zhang, Z. Tang, T. Zhang, H. Xiao, H. Zhuang et al., Electronic modulation and symmetry-breaking engineering of single-atom catalysts driving long-cycling Li−S battery. Angew. Chem. Int. Ed. **64**(6), e202418749 (2025). <https://doi.org/10.1002/anie.202418749>
9. X. Miao, C. Song, W. Hu, Y. Ren, Y. Shen et al., Achieving high-performance lithium–sulfur batteries by modulating Li+ desolvation barrier with liquid crystal polymers. Adv. Mater. **36**(29), 2401473 (2024). <https://doi.org/10.1002/adma.202401473>
10. Y. Jiang, Y. Liao, J. Yu, X. Li, T. Jin et al., Multi-effect ionic liquid additives achieve high cycle stability lithium-sulfur batteries by constructing an electrostatic shielding layer and eliminating ‘dead sulfur’. Adv. Funct. Mater. **35**(32), 2500077 (2025). <https://doi.org/10.1002/adfm.202500077>
